# Lumped‐parameter models of the pulmonary vasculature during the progression of pulmonary arterial hypertension

**DOI:** 10.14814/phy2.13586

**Published:** 2018-02-07

**Authors:** Jesse W. Gerringer, Julie C. Wagner, Daniela Vélez‐Rendón, Daniela Valdez‐Jasso

**Affiliations:** ^1^ Department of Bioengineering University of Illinois at Chicago Chicago Illinois; ^2^ Department of Biomedical Engineering Marquette University Milwaukee Wisconsin; ^3^ Department of Bioengineering University of California at San Diego La Jolla California

**Keywords:** Impedance, mathematical modeling, monocrotaline, parameter estimation, pulmonary arterial hypertension, Windkessel models

## Abstract

A longitudinal study of monocrotaline‐induced pulmonary arterial hypertension (PAH) was carried out in Sprague‐Dawley rats to investigate the changes in impedance (comprising resistance and compliance) produced by elevated blood pressure. Using invasively measured blood flow as an input, blood pressure was predicted using 3‐ and 4‐element Windkessel (3WK, 4WK) type lumped‐parameter models. Resistance, compliance, and inductance model parameters were obtained for the five different treatment groups via least‐squares errors. The treated animals reached levels of hypertension, where blood pressure increased two folds from control to chronic stage of PAH (mean pressure went from 24 ± 5 to 44 ± 6 mmHg, *P* < 0.0001) but blood flow remained overall unaffected. Like blood pressure, the wave‐reflection coefficient significantly increased at the advanced stage of PAH (0.26 ± 0.09 to 0.52 ± 0.09, *P* < 0.0002). Our modeling efforts revealed that resistances and compliance changed during the disease progression, where changes in compliance occur before the changes in resistance. However, resistance and compliance are not directly inversely related. As PAH develops, resistances increase nonlinearly (*R*_d_ exponentially and *R* at a slower rate) while compliance linearly decreases. And while 3WK and 4WK models capture the pressure‐flow relation in the pulmonary vasculature during PAH, results from Akaike Information Criterion and sensitivity analysis allow us to conclude that the 3WK is the most robust and accurate model for this system. Ninety‐five percent confidence intervals of the predicted model parameters are included for the population studied. This work establishes insight into the complex remodeling process occurring in PAH.

## Introduction

Pulmonary arterial hypertension (PAH) is characterized by elevated blood pressure in the pulmonary circulation. During the development of PAH, the pulmonary vasculature undergoes structural remodeling that compromises its normal physiological function. In particular, the pulmonary blood vessels undergo currently untreatable changes and develop lesions that alter their biomechanics and mechanobiology (Stenmark et al. 2009). PAH also subjects the right ventricle of the heart to pressure overload. While cardiac remodeling initially compensates for the increase in pulmonary vascular stiffening, eventually this response becomes maladaptive and, if left untreated, PAH eventually results in cardiac decompensation and heart failure (Vonk‐Noordegraaf et al. 2013). The disease is very aggressive with a 3‐year survival rate of only 48% (Barst 2008) and a prevalence among women is twice as high as in men (Rich et al. 1987). To date, transplantation of the lung remains the only curative treatment option.

Although it has been established that in PAH the pulmonary vasculature stiffens, the causes initiating this remodeling process remain unknown. Clinical studies have shown that pulmonary vascular impedance, a combination of resistance and compliance, is an independent risk factor (Weinberg et al. 2004; Dyer et al. 2006; Stenmark et al. 2009). Thus, it is critical to study the alteration in pulmonary vascular resistance and capacitance to understand the pathophysiology of PAH. Monocrotaline‐induced PAH is a commonly used animal model for the study of this disease. After 4 weeks of being injected with monocrotaline (MCT), the animals reach chronic PAH pressure levels and display vascular lesions (Gomez‐Arroyo et al. 2012; Maarman et al. 2013) such as those found in human PAH patients. Since no longitudinal study of the disease has been carried out, our goal was to characterize the time‐course of changes in vascular resistance and capacitance during the development of the disease in an MCT animal model of PAH. Blood pressure and flow were measured invasively at four stages of the disease. The evolving properties of the pulmonary vasculature were studied by applying the commonly used three‐ and four‐element Windkessel models. Blood flow measurements were used to initiate the Windkessel models, and the resulting blood pressure predictions were compared to the in‐vivo measured data. Model parameters were then used to determine alterations of the resistive and compliant properties of the pulmonary system in the time‐course of PAH. The results showed distinct time‐courses of altered resistance and compliance and will be useful for formulating and testing new computational models.

## Methods & Materials

### Data acquisition

All experimental procedures performed were in accordance with the University of Illinois at Chicago Animal Care and Use Committee. Male Sprague‐Dawley rats (Charles River Laboratories, Chicago, IL) with a mean body weight of 272 ± 30.1 g were included in the study. At 8 weeks of age, the animals were divided into two groups and injected subcutaneously on the posterior aspect of the neck. A group of 27 rats was injected with a dose of 60 mg/kg of monocrotaline (Sigma Aldrich, St Louis, MO, USA) dissolved in HCl and diluted in di‐H_2_O and NaOH at a concentration of 0.025 g/mL. A control group (PL) that accounted for aging effects was injected with saline solution at a volume calculated from the monocrotaline dosage. When possible, a treated (PAH) animal and control (PL) animal were housed together for up to 4 weeks, and underwent a terminal surgical procedure at the end of their specified stage.

**Figure 1:**
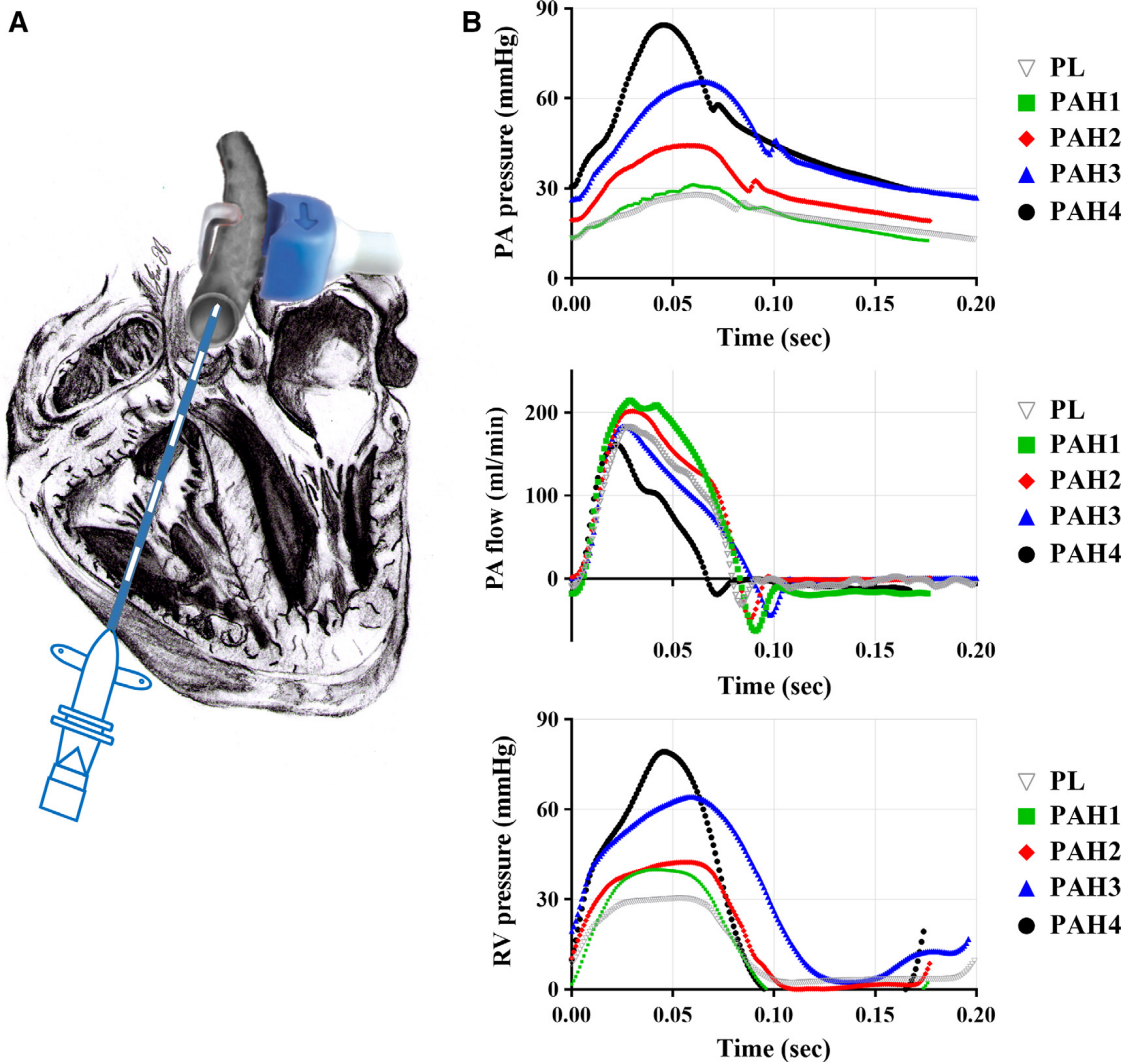
Schematic of in vivo hemodynamic measurements of blood pressure and flow carried out simultaneously. Blood pressure measurements are obtained via a pressure catheter while recording blood flow using an ultrasonic flow probe. (A) Depicts the positioning of the dual pressure sensor catheter and flow probe. (B) Representative MPA blood pressure (top), MPA flow (middle) and right ventricular pressure (bottom) time‐series measurements in normotensive and MCT‐treated animals.

For each week postinjections, paired PL (*N* = 17) and PAH rats underwent an open‐chest surgery to obtain in‐vivo hemodynamic measurements. First, the animals were anesthetized with oxygen at 4% isoflurane, intubated, and placed on a ventilator (SAR‐1000 Ventilator, CWE Inc., Ardmore, PA, USA). The breathing rate was set to 50 breaths/minute and a tidal volume that was based on the animal weight at time of surgery (Pacher et al. 2008). Following a thoracotomy procedure, the heart and large blood vessels were exposed. A 1.6F dual pressure sensor catheter (Transonic Scisense, Ontario, Canada) was inserted into the right ventricle and advanced to the main pulmonary artery (MPA) via the pulmonic valve. An ultrasonic flow probe (Transonic Scisense, Ontario, Canada) was wrapped around the MPA, while continuously measuring blood pressure in the right ventricle and MPA. An illustration of the set‐up for in vivo hemodynamic data measurements is shown in Figure 1A. Time series of pulmonary arterial and right ventricular pressures and blood flow (Fig. 1B) were collected in LabChart software (ADInstruments Inc. Colorado Springs, CO) and further analyzed in a custom‐written MATLAB (version R2016a, The Mathworks, Natick, MA) code for the calculation of hemodynamic parameters such as heart rate, end‐systolic, and end‐diastolic pressures.

### Windkessel models

**Figure 2:**
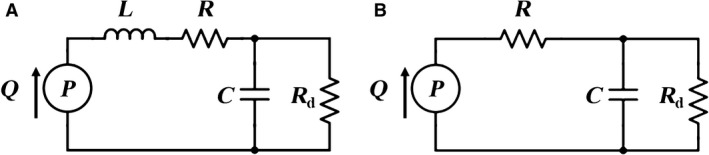
Electrical circuits representing the (A) four‐element Windkessel model (4WK) and (B) three‐element Windkessel model (3WK). The elements in these electrical circuits are inertance (*L*), proximal resistance (*R*), total arterial compliance (C), and distal resistance (*R*_d_). State variables are blood pressure (P) and blood flow (Q).

To identify the individual changes and contributions of resistance and compliance in the vasculature, Windkessel models were used to relate blood flow *Q* to blood pressure *P*. These circuit element‐based models shown in Figure 2 are used as analogies to known physiological properties of the vasculature. In particular, the buffering of pulsatile blood flow performed by the large, proximal vessels can be described through a combination of capacitive, or total vascular compliance (*C*), and proximal resistive (*R*) elements. The function of the distal vessels to convert pulsatile blood flow to steady flow, and thus preventing capillary rupture, can be represented through a highly resistive element (*R*_d_). To describe the inertial aspect of the pulmonary system, an inductor (*L*) circuit element is included to account for the inertial effects of blood. The number of electrical elements used leads to the three‐ or four‐element Windkessel models, with *L* being absent in the three‐element model.

Formulation of the Windkessel equations of blood flow and pressure are based on Kirchhoff's laws. Using Ohm's Law for nodal analysis, the four‐element Windkessel model (Equation 1) relates blood pressure to flow by

(1) $$\;\frac{dP}{dt}+\left(\frac{1}{R_{d}C}\right)P=L\frac{d^{2}Q}{dt^{2}}+\left(R+\frac{L}{R_{d}C}\right)\frac{dQ}{dt}-\left(\frac{1}{C}+\frac{R}{R_{d}C}\right)Q$$

where $\theta_{4\mathrm{WK}}=\left\{R,R_{d},C,L\right\}$ is the set of model parameters. In the absence of inductance, the four‐element model reduces to the three‐element Windkessel model (Equation 2), *viz*.

(2) $$\;\frac{dP}{dt}+\left(\frac{1}{R_{d}C}\right)P=R\frac{dQ}{dt}-\left(\frac{1}{C}+\frac{R}{R_{d}C}\right)Q$$

with the set of model parameters $\theta_{3\mathrm{WK}}=\left\{R,R_{d},C\right\}$.

### Numerical implementation

One cardiac cycle of blood flow was used to predict blood pressure for each Windkessel model. The model parameters *θ*_model_ were estimated via solution of the inverse problem of the predicted and measured values of blood pressure. Starting from a set of initial values *θ*_0_, the inverse algorithm iteratively estimated the set of parameters $\hat{\theta }$ that minimize the normalized root mean square error between the experimental *p*_*j*_ and model predicted *P(t*_*j*_ *,θ)* measured at time points *t*_*j*_. The objective function was defined using a two‐norm error function as shown in Equation 3.

(3) $$\begin{array}{c} E\\ \text{min}\;\theta \end{array}=\sqrt{\frac{\sum_{j=1}^{m}{\left(P\left(t_{j};\theta \right)-p_{j}\right)}^{2}}{\sum_{j=1}^{m}{p_{j}}^{2}}},\text{where}\;j=1,\ldots ,m$$

Here *m* is the number of data points. To numerically implement this objective function, a constrained Nelder–Mead simplex method (*fminsearchbnd* function in MATLAB) was used (D'Errico 2012). This bounded function constrains the parameters to be positive to ensure physical properties (D'Errico 2012). At each iteration of the minimization method, the algorithm solved for pressure *P(t*_*j*_ *,θ)* using a fourth‐order Runge‐Kutta method (*ode45* function in MATLAB). Once a tolerance of 10^−6^ normalized error was met, *fminsearchbnd* function exited, output an optimal set of model parameters $\hat{\theta }$, and convergence of the algorithm was confirmed.

### Initial conditions

The model parameters related to characteristic and total vascular resistance were initialized via input impedance analysis. By mapping three consecutive blood pressure and flow waveforms to the frequency domain using Fourier series, input impedance spectrum (*Z*_in_) for each individual animal was computed (O'Rourke 1982). Briefly, the magnitude of these Fourier coefficients of the pressure and flow signals were then expressed as function of the harmonic term *P(n)* and *Q(n),* respectively. The ratio between *P(n)* and *Q(n)* amplitudes was taken to determine *Z*_in_ as shown in Equation 4. As used in Segers et al., the reflection coefficient (Γ) was computed by taking the following ratio (*Z*_in_ *‐Z*_*c*_ *)/(Z*_in_ *+Z*_*c*_ *)* and the wave‐reflection intensity was determined by evaluating the reflection coefficient (Γ_1_) at the first harmonic or *Z*_*in*_ *(n = 1)* (Segers et al. 1999).

(4) $$Z_{\mathrm{in}}=\frac{P(n)}{Q(n)}$$

Total vascular resistance (*Z*_*0*_ *)* and characteristic impedance (*Z*_*c*_ *)* are metrics based on the impedance magnitude at the 0^th^ order harmonic, and from the average of the higher frequencies from 3rd to 10th harmonics, respectively (Segers et al. 1999). Using these values from each individual animal, the model parameters *R*_d_ and *R* were initialized using the relations $R_{d}^{0}=Z_{o}-Z_{c},R^{0}=Z_{c},$ respectively (Coogan et al. 2011). Inductance was initially set to 0.002 ± 0.001 mmHg·min^2^·mL^−1^ based on work in mice aorta by Segers et al. (2005). Compliance *C*, not being a parameter obtainable from impedance analysis, was determined using the pulse pressure method (PPM) as described by Stergiopulos et al. (1994, 1999). Here, initial compliance is estimated by adjusting the capacitance value of an electrical body (resistor and capacitor in parallel). Blood flow and pressure are used as input and the resistor value is fixed at *Z*_0_. Once the pulse pressure from the model and data match, the value of the capacitor is assigned to the initial compliance *C* ^*0*^. The initial set of parameters *θ*_*0*_ is shown in Table 1.

**Table 1:** Summary statistics of input impedance *Z*_0_, characteristics impedance *Z*_c_, wave‐reflection intensity |Γ_1_|, and arterial compliance based on pressure and flow measurements for each group

Group	*N*	*Z*_0_	*Z*_c_	|Γ_1_|	*C* ^0^
		(mmHg·min/mL)	(mmHg·min/mL)		(mL/mmHg)
PL	17	0.63 ± 0.14^a^^,^ b	0.06 ± 0.02^a^	0.26 ± 0.09^b^	0.32 ± 0.08^a^^,^ b
PAH1	7	0.73 ± 0.26^b^	0.06 ± 0.03	0.32 ± 0.14^b^	0.31 ± 0.12^b^
PAH2	8	0.87 ± 0.33^b^	0.07 ± 0.03	0.32 ± 0.11^b^	0.24 ± 0.11
PAH3	7	1.11 ± 0.24^b^	0.09 ± 0.03	0.36 ± 0.08^b^	0.18 ± 0.09
PAH4	5	1.85 ± 0.59	0.10 ± 0.04	0.52 ± 0.09	0.11 ± 0.03

Based on these values obtained from input impedance analysis and pulse‐pressure method, the model parameters *R*_d_ = *Z*_0_‐*Z*_c_, *R* = *Z*_c_, and *C* were initialized.

a Indicates statistical difference with PAH3.

b Indicates statistical difference with PAH4.

PL (*N* = 17), PAH1 (*N* = 7), PAH2 (*N* = 8), PAH3 (*N* = 7), PAH4 (*N* = 5).

### Sensitivity analysis

To identify the contribution of each model parameter $\hat{\theta_{i}}$ in the prediction of pressure *P(t*_*j*_ *,θ)* for each model, sensitivity analysis was carried out. The sensitivity functions were normalized by the ratio of the parameter and data as shown in Equation 5 (Valdez‐Jasso et al. 2009). To quantify the contribution of each model parameter in the cardiac cycle and numerically rank them, the 2‐norm of the sensitivity equation was computed (Equation 6).

(5) $$\chi_{i}\left(t_{j},{\overset{}{\hat{\theta }}}_{i}\right)=\frac{{\overset{}{\hat{\theta }}}_{i}}{P\left(t_{j};{\overset{}{\hat{\theta }}}_{i}\right)}\frac{dP\left(t_{j};{\overset{}{\hat{\theta }}}_{i}\right)}{d{\overset{}{\hat{\theta }}}_{i}},\text{where}\;j=1,\ldots ,m\;\;\text{and}\;\;i=1,\ldots ,n_{p}$$

(6) $$\left|\right|\chi_{i}\left(t_{j},\overset{}{{\hat{\theta }}_{i}}\right){\left|\right|}_{2},\text{where}\;j=1,\ldots ,m\;\;\text{and}\;\;i=1,\ldots ,n_{p}$$

where *n*_*p*_ is the number of model parameters, and *m* the number of data points in each time series.

### Model selection

The two models were compared using the Akaike Information Criterion (AIC). This criterion allows for model comparison when models contain different number of parameters. The number of parameters used in a model is weighted into the error criterion *E* as follows:(Valdez‐Jasso 2010)

(7) $$\text{AIC}=m\log\left(\frac{E}{m}\right)+2n_{p}$$

### Statistical analysis

Summary statistics were computed for the initial conditions ($R_{d}^{0}=Z_{0}-Z_{c},R^{0}=Z_{c},C^{0}$), hemodynamics and the set of optimized parameters from the Windkessel models (*R*_d_, *R*,* C*,* L*). An analysis of variance (ANOVA) was performed using a Standard Least Squares statistical model to identify differences in means of the parameters according to the disease stage (PL, PAH1, PAH2, PAH3, PAH4). For the univariate recordings of *R*_d_ *, R*, and *C* parameters effects of disease stage (PL, PAH1, PAH2, PAH3, PAH4) and estimation method (3WK, 4WK) were investigated. All the statistical analyses were carried out in JMP Pro Statistical Software (Version 13.0.0, © SAS Institute Inc., Cary, NC). Multiple comparisons were performed using Tukey HSD (Honest Significant Difference) test to determine which differences in average parameter recording among disease stages and estimation methods were significant. For all statistical tests, significance level α was set at 0.05. All values are presented as mean ± standard deviation. Based on the optimized model parameters a 95% confidence interval is included representing each treatment group.

Goodness‐of‐fit was measured via coefficient of determination as shown in Equation 8.

(8) $$R^{2}=1-\frac{SS_{\mathrm{res}}}{SS_{\mathrm{tot}}}$$

where $SS_{\mathrm{tot}}=\sum_{j}{\left(p_{j}-\overset{}{\overset{¯}{p}}\right)}^{2}$, $SS_{\mathrm{res}}=\sum {\left(P_{j}-P\left(t_{j},\theta \right)\right)}^{2}$, $\overset{¯}{p}$ the mean of the observed data *p*_*j*_, *P*(*t*_*j*_, *θ*) the predicted data, SS_tot_ the total sum of squares and SS_res_ the sum of squares of residuals.

## Results

Blood pressure rose in the animals while blood flow remained overall constant. The changes in resistive and compliant properties of the vessels are manifested in the parameter estimation of the 3WK and 4WK model parameters. Blood pressure predictions of the Windkessel models resembled closely the measured pressure. The metrics of model robustness, sensitivity, and accuracy quantified these differences.

### Hemodynamics

**Figure 3:**
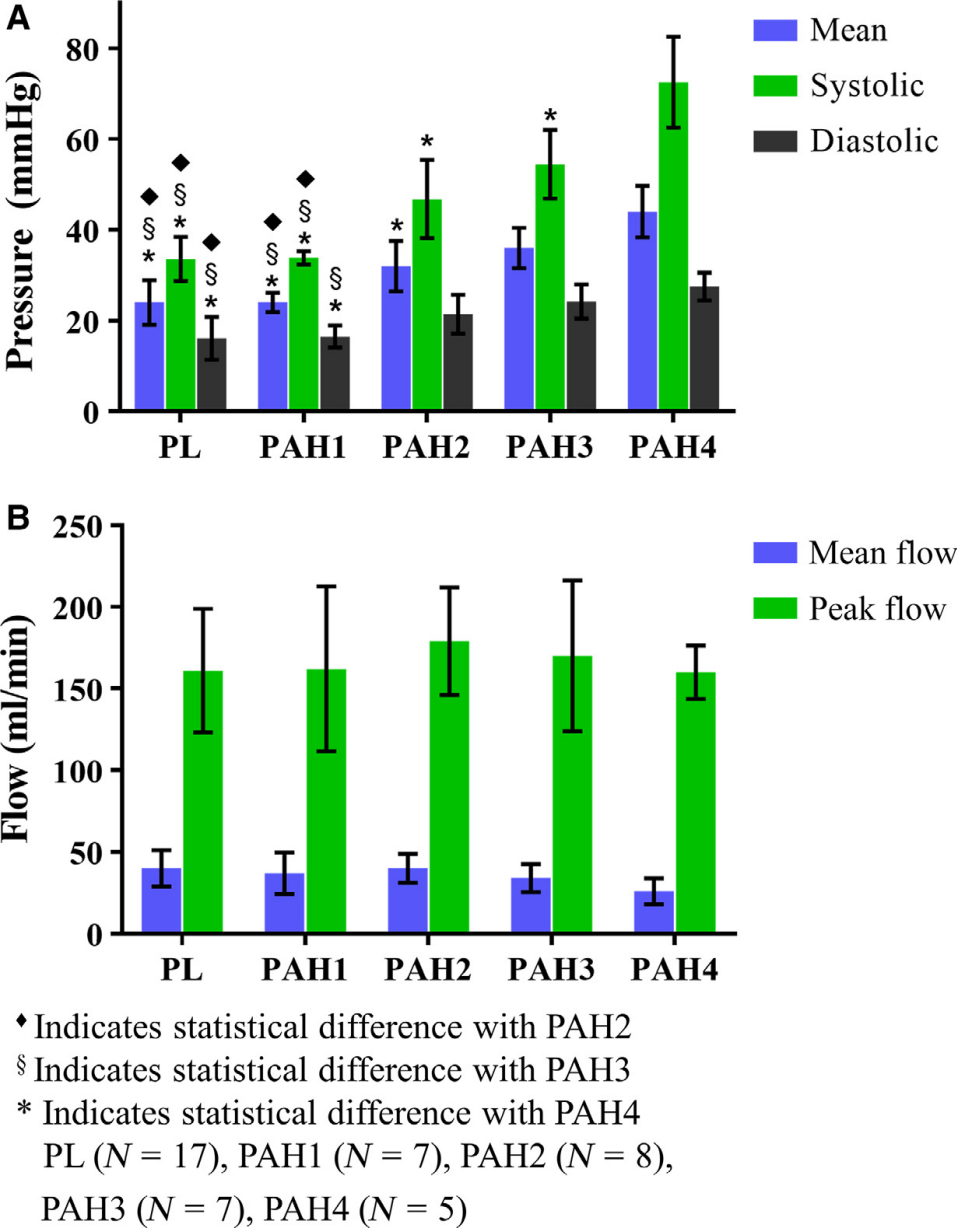
Summary statistics of (A) blood pressure and flow (B) measurements obtained from the main pulmonary artery for the normotensive (PL) and hypertensive (PAH1‐PAH4) groups.

Representative MPA blood pressure (Fig. 1B‐top) and right ventricular pressure (Fig. 1B‐bottom) measurements indicate how the pressure ranges steadily increases as time (weeks) progresses. However, blood flow remains overall constant (Fig. 1B‐middle). The normotensive group had a systolic pressure of 33.6 ± 4.9 mmHg with a mean pulmonary arterial pressure (mPAP) of 24.1 ± 4.9 mmHg, fitting within a normotensive group. In early disease state (PAH1, *N* = 7) the waveform is almost identical to PL (*N* = 17), with a systolic pressure of 33.8 ± 1.5 mmHg and mPAP of 24.7 ± 2.1 mmHg. Between mild and advanced PAH (PAH2, *N* = 7 and PAH3, *N* = 7) the waveforms displayed a sharper increase during systole and a more rapid decrease during diastole coupled with overall increase in mPAP. At the advanced stages of PAH (PAH4, *N* = 5), the systolic pressure increased to 72.5 ± 8 mmHg, with a mPAP of 44.6 ± 5.7 mmHg, an exaggerated phenotype seen in PAH2 (*N* = 8) and PAH3 (*N* = 7). The statistically significant increases in mPAP throughout the different weeks confirmed that MCT induced different stages of PAH. However, the mean flow (or cardiac output) did not show a significant difference among the groups, indicating that the right ventricular performance had not been compromised. The summary statistics of these hemodynamic parameters are presented in Figure 3. Along with the increase in MPA pressure, right ventricular systolic pressure (RVSP) progressively increased with the time course of the disease. Specifically, RVSP_PL_ = 39.7 ± 10.3 mmHg, RVSP_PAH1_ = 35.2 ± 9 mmHg, RVSP_PAH2_ = 44.3 ± 9.2 mmHg, RVSP_PAH3_ = 54.8 ± 10.6 mmHg, and RVSP_PAH4_ = 66.3 ± 9 mmHg.

### Input impedance analysis

The values of *Z*_*0*_, *Z*_*c*_, and *C* ^*0*^ used to initialize *R*_d_, *R*, and *C* per group are presented in Table 1. The normotensive group had a distal resistance (*Z*_*0*_ *‐Z*_*c*_) of 0.57 ± 0.13 mmHg·min·mL^−1^. This differed from the MCT‐treated animals. From the early to advanced stage of PAH (PAH1 to PAH4), *Z*_*0*_ *‐Z*_*c*_ increased from 0.67 ± 0.24 to 1.76 ± 0.60 mmHg·min·mL^−1^ (*P* < 0.0001). This increase was also statistically significant from PL (*N* = 17) group to PAH3 (*N* = 7, 1.02 ± 0.23 mmHg·min·mL^−1^, *P* < 0.0159) and PAH4 (*N* = 5, *P* < 0.0001), from PAH2 (*N* = 8, 0.79 ± 0.31 mmHg·min·mL^−1^) to PAH4 (*N* = 5, *P* < 0.0001), and from PAH3 (*N* = 7) to PAH4 (*N* = 5, *P* < 0.0016). Characteristic impedance *Z*_*c*_ *,* increased at the advanced stages of PAH (*P* < 0.0084), with statistically significant increases from PL (*N* = 17, 0.06 ± 0.02 mmHg·min·mL^−1^) to PAH3 (*N* = 7, 0.09 ± 0.02 Hg·min·mL^−1^) (*P* < 0.0217). The wave‐reflection intensity ¦Γ_1_¦ followed the same trend as the input and characteristic impedance, where it increased from 0.26 ± 0.09 to 0.52 ± 0.09 over the progression of PAH. Statistical significant difference was found for the PAH4 (*N* = 5) group (*P* < 0.0002).

### Model predictions

**Figure 4:**
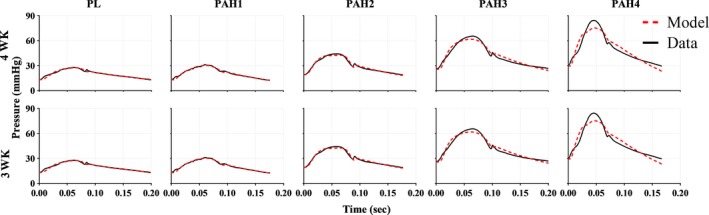
Representative measured (black) and model predicted (red) pulmonary arterial pressure waveforms for the progression of PAH (normotensive to chronic stage). From left to right: Baseline (PL, placebo), early disease stage (PAH1) and far right the chronic disease stage (PAH4). Model predictions based on the (top panel) four‐element Windkessel model (4WK) and (bottom panel) three‐element Windkessel model (3WK).

All the Windkessel models investigated closely predicted the in‐vivo measured blood pressures. Qualitatively, the three‐ and four‐element models predicted blood pressure in the same manner and were able to mimic the dicrotic notch at all the PAH stages, with more accuracy at the early stages of PAH. The measured pressure (solid black lines) and the predicted pressures (dashed red lines) can be visualized in Figure 4. Quantitatively, values of the coefficient of determination *R* ^*2*^ were close to 1.0, and the values in the normalized root mean square error were nearly 0 (Table 2). *R* ^*2*^ values had a decreasing trend with 0.97 in placebos and 0.93 by advanced PAH. It is important to note the *R* ^*2*^ values were identical for the two models. Summary statistics of the optimized set of parameters are shown in Table 2.

**Table 2:** Optimized model parameters, normalized mean square error, and AIC for each model

Model	Group	*R*_d_	*R*	*C*	*L* × 10^−4^	*R* ^2^	Error × 10^−2^	AIC x 10^2^
		(mmHg·min·mL^−1^)	(mmHg·min·mL^−1^)	(mL/mmHg)	(mmHg min^2^ mL^−1^)			
**4WK**	PL	0.57 ± 0.15	0.05 ± 0.02	0.44 ± 0.09	0.26 ± 0.80	0.97 ± 0.01	3.5 ± 0.1	−7.5 ± 1.0
	PAH1	0.66 ± 0.22	0.06 ± 0.03	0.44 ± 0.15	0.15 ± 0.25	0.97 ± 0.01	3.6 ± 0.6	−7.2 ± 0.8
	PAH2	0.78 ± 0.29	0.06 ± 0.02	0.33 ± 0.15	0.02 ± 0.05	0.95 ± 0.03	5.2 ± 1.9	−6.9 ± 1.0
	PAH3	0.96 ± 0.21	0.08 ± 0.04	0.22 ± 0.1	0.04 ± 0.10	0.95 ± 0.02	5.5 ± 0.9	−6.9 ± 0.6
	PAH4	1.60 ± 0.56	0.11 ± 0.03	0.13 ± 0.04	0 ± 0	0.93 ± 0.01	8.1 ± 1.3	−7.4 ± 1.2
**3WK**	PL	0.56 ± 0.14	0.05 ± 0.02	0.43 ± 0.09	–	0.97 ± 0.01	3.6 ± 0.1	−7.5 ± 1.0
	PAH1	0.66 ± 0.22	0.06 ± 0.03	0.43 ± 0.15	–	0.97 ± 0.01	3.6 ± 0.7	−7.2 ± 0.8
	PAH2	0.78 ± 0.29	0.06 ± 0.02	0.33 ± 0.15	–	0.95 ± 0.03	5.2 ± 1.9	−7.0 ± 1.0
	PAH3	0.96 ± 0.21	0.08 ± 0.04	0.22 ± 0.10	–	0.95 ± 0.02	5.5 ± 0.9	−6.9 ± 0.6
	PAH4	1.60 ± 0.56	0.11 ± 0.03	0.13 ± 0.04	–	0.93 ± 0.01	8.1 ± 1.3	−7.4 ± 1.2

PL (*N* = 17), PAH1 (*N* = 7), PAH2 (*N* = 8), PAH3 (*N* = 7), PAH4 (*N* = 5).

### Parameter estimation

**Figure 5:**
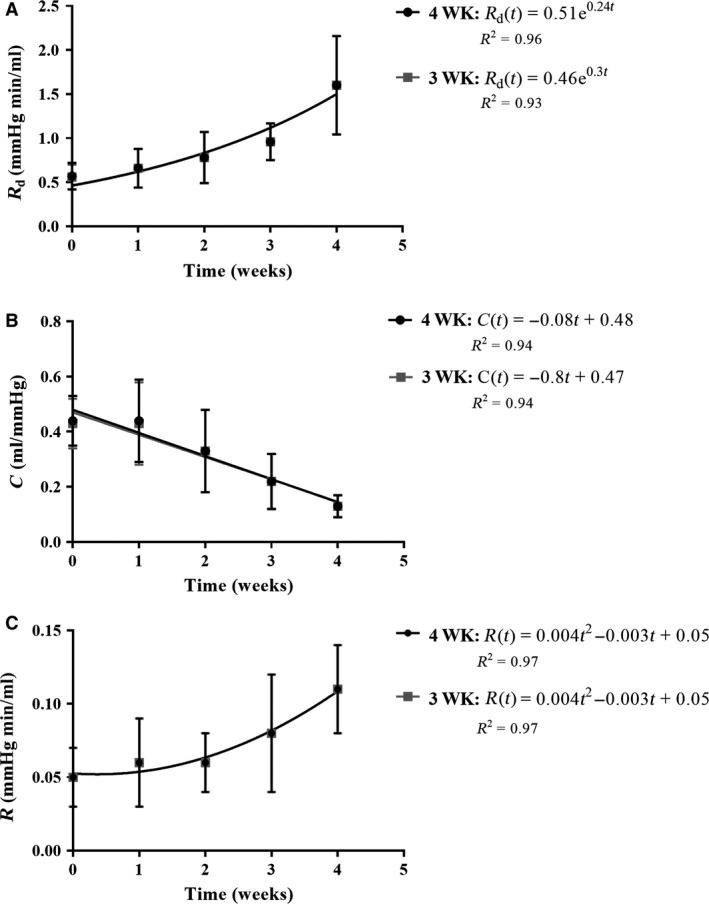
Summary statistics of the parameters estimated via the three‐ (light grey) and four‐element (black) Windkessel model for each treatment group (week 0 corresponds to the placebo group and week 4 the advanced PAH group). Trend‐lines (light grey and black lines) for both models indicate how (A) distal resistance (*R*_d_) increases exponentially while (B) compliance (C) linearly decreases as PAH progresses. (C) Proximal resistance (*R*) increases nonlinearly but at a slower rate. Coefficient of determination *R* ^2^ is included for each model.

The prediction of the parameters *R*_d_, *R*, and *C* using the three‐ and four‐element models was sensitive to the stage of PAH. Both distal and proximal resistances *R*_d_ and *R* nonlinearly increased as the disease progressed in the 3WK and 4WK models (Fig. 5A and C, respectively). Specifically, *R*_d_ exponential increased while *R* nonlinearly rose at a slower rate. On the other hand, the total arterial compliance *C* decreased in a linear fashion (Fig. 5B). Thus, the rate of change in resistances and compliance are not symmetric or proportional to each other. More interestingly, the changes in compliance occur by week 2, earlier than any major changes in resistance (*R* and *R*_d_). This calls to further investigate the mechanisms underlying the remodeling process of the pulmonary vasculature and identify the relation between the resistive and compliant properties of this nonlinear system.

*R*_d_ was statistically larger in the later stages of PAH compared to the normotensive state. In the models, *C* significantly decreased from normotensive to the chronic stage of PAH. The parameter *R* displayed a pattern like *R*_d_, but leveled off at weeks 3 and 4 with a significant increase from PL (*N* = 17) and PAH2 (*N* = 8) to PAH4 (*N* = 5). Lastly, the inductor *L* from the 4WK model varied across the PL (*N* = 17) and treated MCT groups with PAH4 (*N* = 5) ranging close to zero. The parameter L was transformed using the natural logarithm, ln(L), in an effort to detect statistical differences, however, there was not a significant change in the parameter with PAH progression (*P* < 0.3834). Method predictions were not statistically significant different between 3WK and 4WK for none of the parameters (*R*_d_: *P* < 0.9698, *R*:* P* < 1.0000, *C*:* P* < 0.8775). *P*‐values for treatment and method effects are summarized in Table 3, where the values less than 0.05 were considered statistically significant.

**Table 3:** *P*‐values for treatment and method effects in the Windkessel parameters (*R*_d_ *, R, C,* and *L*)

Parameter/effect		Treatment		Method
*R*_d_ (mmHg·min·mL^−1^)		*P* < 0.0001		*P* < 0.9698
	PAH0‐PAH3		*P* < 0.0003	None
	PAH0‐PAH4		*P* < 0.0001	
	PAH1‐PAH3		*P* < 0.0461	
	PAH1‐PAH4		*P* < 0.0001	
	PAH2‐PAH4		*P* < 0.0001	
	PAH3‐PAH4		*P* < 0.0001	
*R* (mmHg·min·mL^−1^)		*P* < 0.0001		*P* < 1.0000
	PAH0‐PAH4		*P* < 0.0001	None
	PAH1‐PAH4		*P* < 0.0014	
	PAH2‐PAH4		*P* < 0.0003	
*C* (mL/mmHg)		*P* < 0.0001		*P* < 0. 8775
	PAH0‐PAH2		*P* < 0.0193	None
	PAH0‐PAH3		*P* < 0.0001	
	PAH0‐PAH4		*P* < 0.0001	
	PAH1‐PAH3		*P* < 0.0001	
	PAH1‐PAH4		*P* < 0.0001	
	PAH2‐PAH4		*P* < 0.0007	
ln(*L*)(mmHg·min^2^·mL^−1^)		*P* < 0.3834		N/A
	None			

Statistical significance is considered when *P* < 0.05. Treatment had significant additive effect on the *R*_d_ *, R,* and *C*. Method did not show any difference between the 3‐ and 4‐element models.

### Sensitivity analysis

The ranking of the optimized *R*_d_, *R*, and *C* parameters were on the same order of magnitude. *R*_d_ had the largest magnitude value, almost twofold higher than the other parameters indicating that *R*_d_ is the biggest contributor in the model. This was followed by the compliance parameter *C* and *R*. Specifically, in the 3WK model *R*_d_ rank ranged between 5.4 ± 0.8 and 8.3 ± 0.8, whereas *C* from 2.6 ± 0.2 to 3.7 ± 0.3, and R from 1.6 ± 6.1 to 2.0 ± 6.7, respectively (Table 4). The *R*_d_ and *C* parameters had the highest numerical ranking and similar values across both Windkessel models and their relative contribution to the models remained unaffected by the disease. For the four‐element Windkessel models, *L* was two orders of magnitude lower than the rest of the parameters. Due to this large difference in magnitude and thus low ranking, this indicated the contribution of *L* to be significantly smaller than the other parameters. In this case, the contribution of the inductor *L* to the model prediction can be neglected.

**Table 4:** Numerical ranking of the model parameters based on sensitivity analysis

Model	Group	*R*_d_	*R*	*C*	*L*
4WK	PL	5.8 ± 0.8	2.0 ± 6.7	2.9 ± 0.2	0.01 ± 0.01
	PAH1	5.3 ± 0.8	2.0 ± 5.5	2.6 ± 0.2	0.05 ± 0.07
	PAH2	6.2 ± 1.0	1.8 ± 5.2	2.9 ± 0.4	0.01 ± 0.02
	PAH3	7.1 ± 1.6	1.6 ± 6.1	3.1 ± 0.4	0.01 ± 0.02
	PAH4	8.3 ± 0.8	2.0 ± 3.1	3.7 ± 0.3	0 ± 0
3WK	PL	5.8 ± 0.8	2.0 ± 6.7	2.9 ± 0.2	–
	PAH1	5.4 ± 0. 8	2.0 ± 5.4	2.6 ± 0.2	–
	PAH2	6.3 ± 1.0	1.8 ± 5.2	2.9 ± 0.4	–
	PAH3	7.1 ± 1.6	1.6 ± 6.1	3.1 ± 0.4	–
	PAH4	8.3 ± 0.8	2.0 ± 3.1	3.7 ± 0.3	–

The distal resistance *R*_d_ had the highest contribution, followed by *C* and *R* for both Windkessel models. *L* contributed the least in the 4WK with two orders of magnitude smaller.

PL (*N* = 17), PAH1 (*N* = 7), PAH2 (*N* = 8), PAH3 (*N* = 7), PAH4 (*N* = 5).

### Model selection

Normalized root mean square error *E* and the AIC values were used to determine the model closest to the data while accounting for the number of parameters used. As shown in Table 2, E and AIC values for 4WK and 3WK are within the same ranges for each stage of PAH. The cost of adding a fourth parameter was, therefore, incremental. Furthermore, confidence interval results show the parameters, excluding *L*, initially have a relative narrow range (Table 5). As the disease progresses, these intervals become larger, which may be attributed to the less accurate fitting of the severe PAH conditions. *L* was shown not to statistically change over the progression of PAH and the confidence intervals further reflect the low contribution of this parameter to the model predictions.

**Table 5:** Confidence intervals for the estimated model parameters for the three‐ and four‐element Windkessel models for a male Sprague‐Dawley rat population of MCT‐induced PAH

Model	Group	*R*_d_ (mmHg·min·mL^−1^)	*R* (mmHg min mL^−1^)	*C* [mL/mmHg]	ln(L) (mmHg·min^2^·mL^−1^)
4WK	PL	[0.49, 0.64]	[0.4, 0.7]	[0.39, 0.49]	[−19.00, −14.059]
	PAH1	[0.44, 0.88]	[0.3, 0.9]	[0.29, 0.59]	[−18.94, −11.10]
	PAH2	[0.52, 1.04]	[0.4, 0.8]	[0.19, 0.46]	[−21.38, −14.66]
	PAH3	[0.75, 1.17]	[0.4, 1.2]	[0.13, 0.32]	[−22.94, −15.15]
	PAH4	[0.83, 2.37]	[0.8, 1.5]	[0.07, 0.19]	[−20.22, −16.66]
3WK	PL	[0.49, 0.64]	[0.4, 0.7]	[0.39, 0.48]	–
	PAH1	[0.43, 0.88]	[0.3, 0.9]	[0.29, 0.59]	–
	PAH2	[0.52, 1.04]	[0.4, 0.8]	[0.19, 0.46]	–
	PAH3	[0.75, 1.17]	[0.4, 1.2]	[0.13, 0.32]	–
	PAH4	[0.83, 2.37]	[0.8, 1.5]	[0.07, 0.19]	–

PL (N = 17), PAH1 (N = 7), PAH2 (N = 8), PAH3 (N = 7), PAH4 (N = 5).

## Discussion

In this study, pulmonary vascular remodeling properties were investigated in an animal model of PAH. Using lumped‐parameter Windkessel models, in‐vivo measurements from the main pulmonary artery were used to identify changes in vascular resistance, characteristic impedance, vascular compliance, and inertial effects at each stage of the disease. As blood pressure increased in the pulmonary system, the resistive (*R* and *R*_d_) and compliant (*C*) properties of the vessels changed, compliance changing earlier in the time‐course. Inductance contributions were not significant in the 4WK model. Outcomes from AIC and sensitivity analysis indicate that the 3WK is sufficient to represent the impedance characteristics of the pulmonary system during the progression of PAH in the MCT‐animal model.

### Parameter estimation

The computed initial conditions followed the expected behavior and provided with a good starting point for the optimization process. Several studies have shown a significant increase in total vascular resistance (*Z*_*0*_) with increased afterload in both systemic and pulmonary systems (Murgo et al. 1980; Segers et al. 2005; Hunter et al. 2008). Our findings showed this trend from normotensive to chronic disease state, as well as the significant increases during the disease progression. The average of the higher harmonics, represented by the characteristic impedance *Z*_c_, also tended to increase, albeit less significantly. As expected, our initial *C* ^*0*^ was lower in value compared to the optimized parameter *C*, as has been seen previously by Segers et al. (1999). This was attributed to the pulse‐pressure method focusing on lower harmonics, which is where compliance is mostly affected, therefore possibly giving a more accurate representation (Segers et al. 1999). Overall, decreases in *C* from week to week in each model indicate a stiffening of the pulmonary vasculature (Thenappan et al. 2016), which has also been observed in the systemic circulation (Shirwany and Zou 2010; Wu et al. 2015). The impedance values *Z*_*0*_ *‐Z*_*c*_ and *Z*_*c*_ differed and were underestimated from those based on the parameter estimation routine. This can be explained as the model parameter *C* is not identified via input impedance analysis, overestimating *R*_d_ and *R*. Finally, the wave‐reflection intensity increased progressively with the disease. This parameter is important in confirming the change in overall stiffness in the pulmonary vasculature as the disease progressed. As PAH becomes more severe, the wave‐reflection intensity, and therefore stiffness in the vasculature increased (Nichols et al. 2008).

As PAH progressed, the optimized parameters followed the same trend as the initial conditions. The differences, however, were that the optimized parameters were able to distinguish the unique contribution of resistance and compliance of the vasculature. More importantly, the optimized model parameters offered a mechanistic understanding of the system allowing for a more physiologically accurate representation. It was observed that the more severe PAH is, the stiffer the vasculature becomes, primarily because of decreased *C* (Thenappan et al. 2016). More specifically, the rate of increase in the resistance is not proportional to the rate of decrease in compliance. Also, changes in compliance occur before major changes in resistance. This indicates an inverse nonlinear relationship between the two parameters and further modeling efforts are needed to address the underlying mechanism of pulmonary vascular remodeling during PAH. To the best of our knowledge, the outcomes presented in this study are the first to indicate how the resistance and compliant properties are changing as a function of the PAH progression in the pulmonary vasculature. Lankhaar et al. (2006) conducted a study with human patients with different severities of PAH (chronic thromboembolic and idiopathic), however, this does not display how the severity changes as a function of disease in a specific individual.

In a week‐to‐week comparison, *R*_d_ follows an exponential increase as the disease progresses, whereas *C* follows a linear decrease. In fact, sooner changes in compliance can be attributed to an early remodeling process occurring in the larger vessels instead of the resistive vessels. Due to the models being a nondimensional model, the exact interpretation of these results is limited (Westerhof et al. 2009). Future work will involve the development of a one‐dimensional fluid model in order to incorporate wall shear stress and add spatial components to determine the locations of where resistance and compliance change in the vasculature. This would reveal whether the behavior of the proximal vessels during disease progression imposes the behavior on the distal vessels, or vice versa.

### Model selection

Both models described the physiological system by accurately predicting the dicrotic notch in the pressure waveform, as well as providing physiologically realistic values. However, given that the 3WK model offers the same evaluation than the 4WK does, including inductance for the modeling of PAH impedance is not necessary. In work done by Lambermont et al. (1997) the inclusion of *L* was important to improve their data fitting but they did not evaluate the contribution of the inductor through a sensitivity analysis. Our findings show, however, that the contribution of the inductor in the four‐element model is negligible. In addition, the large confidence interval of *L* further confirms the uncertainty of this parameter to model the pulmonary vascular hemodynamics. We suggest that the three‐element Windkessel model is sufficient to accurately and robustly represent the pulmonary system dynamics.

While both models predicted the blood pressure, the model‐to‐data discrepancy increased as PAH progresses. We speculate that the pressure waveforms may become more complex as the disease progresses and the Windkessel models may be limited to capture these changes in hemodynamics. This in turn motivates the development of a 1D fluid model to more accurately capture pulmonary vascular remodeling in PAH.

To summarize, we found that the three‐element Windkessel model organized in series provided accurate and physiological predictions for resistive and compliant properties in the pulmonary vasculature. It is evident that with the progression of PAH, total vascular resistance is increasing exponentially, while the overall compliance has a steady, linear decrease. Furthermore, more pronounced changes are occurring earlier in compliance. The interaction of the resistant and compliant properties may give insight into where and how the pulmonary vasculature is remodeling as a function of disease state and be useful in future modeling (i.e., 1D fluid model) of PAH. More specifically, based on where the linear compliant and nonlinear resistant changes are occurring, targeted treatment methods could be devised to mitigate the issues in those regions.

### Limitations

The main limitations of this model were the lack of spatial representation of the pulmonary system as well as lack of wave transmission information. In the future, with proper data acquisition, this model can be incorporated as a boundary condition for a one‐dimensional fluid model to give understanding of the complex properties of the pulmonary vasculature as it undergoes PAH progression. In addition, the study was only limited to male rats. Even though the incidence of PAH in women is almost twice as in men (Foderaro and Ventetuolo 2016), a study by Ventetuolo et al. (2014) reported that female patients have a better survival than male patients. Furthermore, while PAH incidence is higher in females than in males, rodent models of PAH have suggested that estrogen play an important role in the pulmonary vasculature, producing beneficial effects in response to PAH (Pugh and Hemnes 2010). Future work will study how compliance and resistance change over the disease progression in female rats.

## Conflict of Interest

None declared.

## Data Accessibility
